# Combined lipid emulsion and plasma exchange in massive pediatric bupropion overdose: Case Report

**DOI:** 10.3389/ftox.2025.1738642

**Published:** 2026-01-08

**Authors:** Brett Russell, Michael Reedy, Kristina M. Flathers, Christine M. Matula, Erica Stevens, Friederike Strelow, Yosef Levenbrown

**Affiliations:** 1 Pediatric Critical Care, Nemours Children’s Health, Wilmington, DE, United States; 2 Pharmacy Services, Nemours Children’s Health, Wilmington, DE, United States; 3 Medical Library, Nemours Children’s Health, Wilmington, DE, United States; 4 Extracorporeal Membrane Oxygenation, Nemours Children’s Health, Wilmington, DE, United States; 5 Apheresis, Nemours Children’s Health, Wilmington, DE, United States; 6 Pediatric Cardiac Critical Care, Nemours Children’s Health, Wilmington, DE, United States

**Keywords:** adolescent, bupropion, cardiac arrest, extracorporeal membrane oxygenation, overdose

## Abstract

**Introduction:**

Massive bupropion overdose in pediatric patients can result in refractory cardiogenic shock, seizures, and cardiac arrest requiring aggressive intervention. Although lipid emulsion (LE), therapeutic plasma exchange (TPE), and extracorporeal membrane oxygenation (ECMO) have been used, evidence guiding clinical management remains limited. We present a case of combined LE therapy and TPE in a pediatric patient with massive bupropion overdose.

**Methods:**

An adolescent female with intentional ingestion of 27,000 mg bupropion XL and 2,250 mg metoprolol XL experienced cardiac arrest requiring veno-arterial ECMO. Lipid emulsion therapy was followed by serial therapeutic plasma exchange. Serial urine samples quantifying bupropion and hydroxybupropion elimination were analyzed.

**Results:**

Following treatment with LE and plasma exchange, the patient rapidly improved clinically, with declining urine concentrations of bupropion and hydroxybupropion showing enhanced drug clearance.

**Conclusion:**

This case supports using combined LE therapy and TPE to enhance drug elimination and improve outcomes in massive pediatric bupropion overdose.

## 1 Introduction

Bupropion hydrochloride is an aminoketone class antidepressant medication with indications for use including major depressive disorder, seasonal affective disorder, and smoking cessation along with off-label usage for attention-deficit/hyperactivity disorder, generalized anxiety disorder, and bipolar disorder. Bupropion is hepatically metabolized and renally excreted, has a prolonged half-life, and exhibits linear kinetics (Farias-Moeller and Carpenter, 2017). Bupropion is metabolized to three key metabolites, hydroxybupropion, erythrohydrobupropion, and threohydrobupropion, with hydroxybupropion being the most notable (Al-Abri et al., 2013). The half-life of bupropion and hydroxybupropion ranges from 20 to 37 h under normal elimination circumstances (Vilayet et al., 2024). The mechanism of action of bupropion’s neurochemical effect is not well known but has weak inhibition of reuptake of dopamine and norepinephrine (Rianprakaisang et al., 2021). Supratherapeutic levels of bupropion have been reported to cause seizures, dysrhythmias, and cardiovascular collapse resulting in cardiac arrest (O’Brien et al., 2021). This could be significantly prolonged with ingestion of supratherapeutic doses, as in the case of an intentional overdose.

We present the case of an adolescent female with intentional co-ingestion of 27,000 mg of bupropion XL and 2,250 mg of metoprolol XL, resulting in cardiac arrest and subsequent initiation of veno-arterial extracorporeal membrane oxygenation (VA-ECMO). We describe the impact of lipid emulsion therapy paired with plasma exchange on the elimination of bupropion and its active metabolite, hydroxybupropion, as measured by serial creatinine adjusted urine samples (to account for variability in hydration status and effect it would have on urine drug levels). Secondary outcomes include clinical improvements in cardiovascular, neurological, and renal function following these interventions.

## 2 Case summary

An adolescent female was brought to the emergency department of a partner hospital by emergency medical services after being found unresponsive by family following an intentional overdose of 27,000 mg of bupropion XL and 2,250 mg of metoprolol XL. En route, she experienced a seizure that was terminated with benzodiazepines. She was hypotensive (systolic blood pressure 60–70 mmHg) and hypoventilating, necessitating positive pressure ventilation. Upon emergency department arrival, she was intubated, central venous access was established, and vasoactive support was initiated. She received multiple doses of glucagon for bradycardia, followed by a continuous infusion. A norepinephrine infusion was started for persistent hypotension. On examination, she had fixed, dilated pupils and absent cough and gag reflexes.

Upon arrival to the pediatric intensive care unit (PICU), the patient remained hypotensive (blood pressure 60–70s/50 s mmHg), requiring ongoing norepinephrine and epinephrine infusions. Glucagon infusion was continued to address bradycardia and hypotension due to beta-blocker toxicity. Calcium gluconate was administered for refractory hypotension. The patient demonstrated poor neurologic function, with 8 mm nonreactive pupils, no spontaneous respiratory effort in the absence of sedation, and absent cough and gag reflexes. Initial electrocardiogram revealed a QTc of 510 ms. Magnesium sulfate was administered for a serum magnesium level of 1.8 mg/dL.

In consultation with a poison control toxicologist, high-dose insulin therapy was initiated for refractory beta-blocker toxicity. Intravenous lipid emulsion (LE) was also administered as a “lipid sink” to mitigate potential cardiotoxicity from bupropion. Despite escalating vasoactive support, the patient developed worsening hypotension and experienced a sudden transition to pulseless wide complex tachycardia. Cardiopulmonary resuscitation was initiated, and the patient underwent three unsuccessful defibrillation attempts. A total of 11 doses of intravenous epinephrine and lidocaine were administered. Due to failure to achieve return of spontaneous circulation (ROSC), the patient was cannulated for VA-ECMO. Total cardiopulmonary resuscitation duration prior to and during extracorporeal membrane oxygenation (ECMO) initiation was 64 min.

Following ECMO initiation, the patient was comatose, with dilated nonreactive pupils, absent cough and gag reflexes, and no spontaneous respirations despite being off sedatives and analgesics. An echocardiogram performed 2 h after ECMO initiation showed severely depressed biventricular function with an ejection fraction of 18%. The patient had no palpable pulses and was fully dependent on ECMO for circulatory support. Phenylephrine and vasopressin infusions were started to maintain a target mean arterial pressure of 65 mmHg. She developed oliguric acute kidney injury secondary to cardiac arrest and fluid overload necessitating initiation of continuous renal replacement therapy (CRRT).

A 50-g dose of activated charcoal was administered via nasogastric tube for potential adsorption of residual drug in the gastrointestinal tract, followed by whole bowel irrigation with polyethylene glycol. After an initial 20% intralipid bolus (1.5 mL/kg), a continuous infusion was initiated at 0.025 mL/kg/min for 24 h. High-dose insulin therapy was continued for beta-blocker toxicity. Despite these interventions, the patient’s condition showed no improvement. Approximately 18 h after admission, she developed seizures and was placed on continuous electroencephalography (EEG) monitoring. Initial EEG showed a burst suppression pattern with prolonged intervals and episodes of non-convulsive seizures. Seizures were treated with lorazepam followed by a loading dose of levetiracetam and transition to twice-daily maintenance dosing.

On hospital day 2, therapeutic plasma exchange (TPE) was initiated to rapidly reduce bupropion levels. Prior to each exchange, LE therapy was administered to mobilize bupropion into the intravascular compartment and enhance clearance via TPE. The patient received an intralipid infusion at 200 mL/h for 3 h immediately before each exchange and underwent daily 1.5-volume plasma exchanges for five consecutive days. Due to the high exchange volumes and concurrent heparin anticoagulation for ECMO, plasma was used as replacement fluid to preserve coagulation factor levels.

**FIGURE 1:**
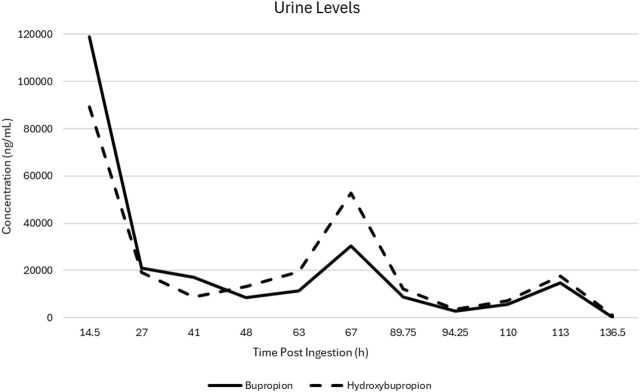
Urine bupropion and hydroxybupropion drug levels following initiation of plasma exchange and lipid emulsion therapies.

Urine levels of bupropion and hydroxybupropion were measured before and after each plasma exchange (see Table 1; Figure 1). Initial concentrations were 119,000 ng/mL and 89,200 ng/mL, respectively, which dropped markedly to 20,900 ng/mL and 19,300 ng/mL following the first exchange. After 2 days without clinical improvement, the patient demonstrated significant neurologic and cardiovascular recovery following the initial plasma exchange. For the first time since VA-ECMO initiation, she developed a pulse pressure of 20–30 mmHg, and echocardiography showed improved biventricular function with ejection fraction increasing from 18% to 33%. Neurologic improvement was noted, too, as pupils became reactive, cough reflex returned with endotracheal suctioning, and spontaneous extremity movements were observed. Also, EEG findings improved from burst suppression to diffuse delta slowing with no seizure activity. Sedation was initiated at that point due to increased alertness.

**TABLE 1:** Urine bupropion and hydroxybupropion levels before and after each plasma exchange.

Time post ingestion (h)	Post PLEX sample timing (min)	Bupropion (ng/mL)	Hydroxybupropion (ng/mL)	Percent change (BP/HB)
14.5	N/A	119,000	89,200	−82.4/-78.4
27	108	20,900	19,300	
41	N/A	17,000	8,870	−50.6/+48.8
48	68	8,400	13,200	
63	N/A	11,400	19,500	+167.5/+170.8
67	17	30,500	52,800	
89.75	N/A	8,800	12,100	−68.2/-70.8
94.25	218	2,800	3,530	
110	N/A	5,590	7,230	+166.5/+143.4
113	10	14,900	17,600	
136.5^a^	N/A	296	1,070	N/A

^a^ Level obtained 23.5 h post-PLEX5. x: half-life not calculated due to increasing levels or inappropriate sample timing. BP, bupropion; HB, hydroxybupropion; N/A, not obtained.

On hospital day 3, the second plasma exchange commenced with continued clinical improvement. The patient tolerated weaning of inotropic support, as well as weaning of ECMO support from 3.5 to 2.7 L per minute, and fluid removal was able to take place through CRRT without resulting in hypotension.

Following the third plasma exchange, the patient demonstrated marked improvement in cardiac function, with echocardiography showing normal biventricular function and an ejection fraction of 50%. She was subsequently decannulated from ECMO, and CRRT was discontinued. Vasoactive support was weaned to minimal phenylephrine doses. Neurologic status continued to improve. Daily intralipid infusions followed by plasma exchange were continued for two additional days post-ECMO decannulation due to persistent encephalopathy and elevated bupropion and hydroxybupropion levels.

**FIGURE 2:**
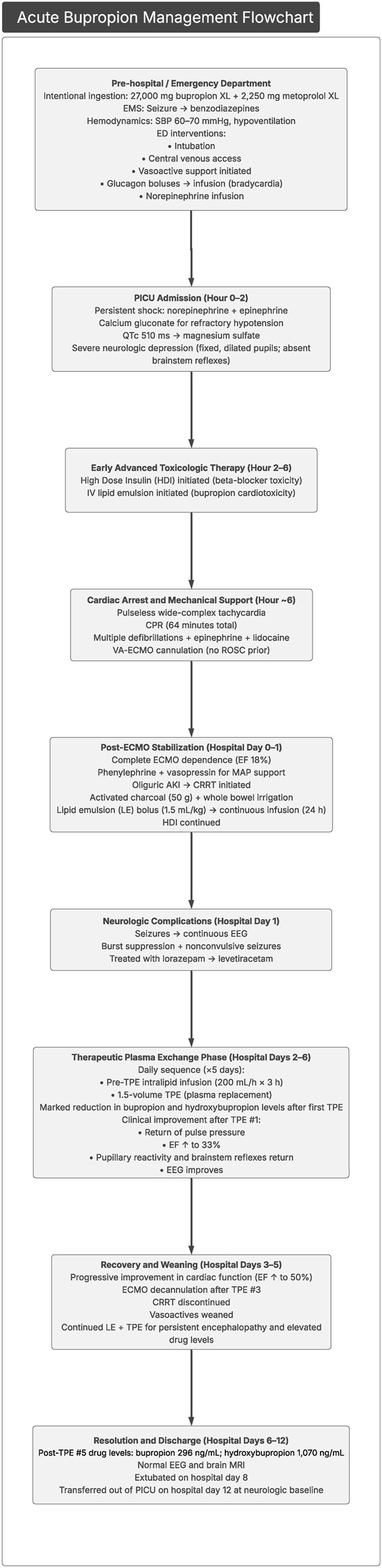
Flowchart highlighting the key features of this case presentation. CRRT, continuous renal replacement therapy; ECMO, extracorporeal membrane oxygenation; EEG, electroencephalography; EF, ejection fraction; HDI, high-dose insulin; MAP, mean arterial pressure; MRI, magnetic resonance imaging; PICU, pediatric intensive care unit.

Approximately 24 h after the fifth plasma exchange, urine bupropion and hydroxybupropion levels had fallen to 296 and 1,070 ng/mL, respectively, with continued clinical improvement. A follow-up EEG was performed and was normal. A brain magnetic resonance image was completed and was normal, without evidence of hypoxic ischemic changes or other intracranial abnormalities. On hospital day 8, the patient was successfully extubated with intact neurological function. She was transferred out of the PICU on hospital day 12 with baseline mentation. The key features in this case presentation are summarized in Figure 2.

## 3 Discussion

The toxicity seen with bupropion overdoses ranges from mild to life-threatening. Mild to moderate symptoms include agitation, hallucinations, tachycardia, tremor, and single seizure. Severe symptoms include hypotension, multiple seizures, QRS widening, QTC prolongation, status epilepticus, and ventricular dysrhythmias. Bupropion overdose commonly results in tachycardia and more rarely conduction disturbances. Rarely, severe toxicity can cause QT prolongation and QRS widening (Brown and Crouch, 2017). Tachycardia serves as the most sensitive predictor of seizure risk, with 93% of patients without tachycardia remaining seizure-free during observation. The drug’s large volume of distribution (20–30 L/kg) and hepatic metabolism render conventional therapies like hemodialysis ineffective, necessitating alternative interventions (Brown and Crouch, 2017).

Overdose of bupropion with subsequent therapeutic support has been reported in several case studies regarding ECMO for cardiovascular collapse as well as use of LE therapy to assist in drug clearance (Brown and Crouch, 2017; Bornstein et al., 2019; Bucklin et al., 2013; Heise et al., 2016; Shenoi et al., 2011; Smolinske et al., 2019). No studies have been published regarding the administration of LE therapy in conjunction with plasma exchange, nor on the impact of plasma exchange on pharmacokinetics and clinical outcomes in cases of severe bupropion overdose. This current report is the first to demonstrate the effect that daily LE therapy followed by plasma exchange has, both on a patient’s clinical course as well as drug levels, following a massive bupropion overdose. Obtaining bupropion and hydroxybupropion levels before and after each plasma exchange also allowed us to monitor the trend in urine concentrations.

The published literature describing management of massive, life-threatening bupropion overdose is limited to case reports and small observational analyses. Prior reports primarily describe the use of LE therapy and ECMO in patients with refractory neurologic or cardiovascular instability.

Four studies report LE use in severe bupropion toxicity. A toxicology database analysis including 53 bupropion overdoses identified five cases in which LE administration was associated with ROSC among 34 total ROSC events treated with intralipid therapy (Smolinske et al., 2019). Case-level reports provide more detail regarding timing, dosing, and response. In one pediatric case of massive bupropion XR ingestion (24–133 mg/kg) complicated by seizures, conduction abnormalities, and persistent encephalopathy, a 1.5 mL/kg bolus of 20% LE administered on intensive care unit day 1 resulted in marked improvement in mental status within 30 min. Recurrent neurologic deterioration on day 3 responded similarly to repeat dosing at the same bolus, with sustained resolution of symptoms (Bornstein et al., 2019). In another report of combined bupropion and lamotrigine overdose complicated by cardiac arrest, a 100 mL bolus of 20% LE restored palpable pulses within approximately 1 min, followed by QRS narrowing, restoration of sinus rhythm, and reduced vasopressor requirements over the subsequent 15 min (Sirianni et al., 2008).

In contrast, two reports describe limited efficacy of LE in patients who ultimately required ECMO support. In one case, a 1.5 mL/kg bolus of 20% lipid emulsion produced no appreciable hemodynamic response prior to initiation of VA-ECMO (Heise et al., 2016). In another, administration of a substantially higher dose (20 mL/kg) failed to prevent progression to refractory shock requiring ECMO (Shenoi et al., 2011).

ECMO has emerged as a rescue therapy for refractory cardiogenic shock due to massive bupropion overdose. Two reports describe three patients supported with VA-ECMO for cardiogenic shock or cardiac arrest unresponsive to multiple vasopressors. All patients survived to discharge with full recovery. Duration of VA-ECMO support ranged from 71 h to 4 days; one patient required an additional 6 days of VV-ECMO for persistent respiratory failure, for a total ECMO duration of 10 days (Heise et al., 2016; Shenoi et al., 2011).

Drugs with high plasma protein binding affinity (>80%) remain largely confined to the intravascular compartment and are most susceptible to removal during plasma exchange. When volume of distribution exceeds 0.2 L/kg, drugs distribute extensively into tissues beyond the plasma compartment, reducing the effectiveness of plasma-based removal techniques (Cheng et al., 2017; Jones and Dougherty, 1986). Although bupropion has moderate protein binding of 84%, its large volume of distribution of 40 L/kg (French et al., 2011) makes the drug difficult to eliminate with plasma exchange alone. As such, we utilized LE to draw the drug and prodrug into the plasma prior to each plasma exchange. This was followed by daily 1.5 volume plasma exchange with fresh frozen plasma for a total of five treatments.

Multiple theories have been proposed to explain the mechanism of lipid emulsion therapy. The static lipid sink theory, suggests that the lipid phase of the emulsion sequesters lipophilic drugs from highly perfused organs such as the heart and brain. According to this theory, LE therapy rapidly creates an expanded lipid compartment within the intravascular space, drawing drug molecules off cellular receptors and into the plasma, trapping them in the lipid phase. In a case report involving a massive bupropion overdose, the patient’s serum bupropion level peaked shortly after lipid administration–supporting the presence of a lipid sink effect. Furthermore, the bupropion concentration declined in parallel with serum triglyceride levels, while no such change was observed for lamotrigine, a drug with low lipophilicity. The same report also noted rapid reversal of cardiotoxic effects following lipid administration–an observation consistent with the clinical course in our patient (Sirianni et al., 2008; Hwang and Sohn, 2024).

A second proposed mechanism is the *lipid shuttle theory*. This theory suggests that LE creates multiple lipid compartments within the bloodstream, which absorb lipophilic drugs from highly perfused organs such as the heart and brain. These lipid-bound drug complexes are then redistributed to tissues with lower perfusion, such as skeletal muscle, liver, and adipose tissue, where the drugs can be sequestered, metabolized, or detoxified (Sirianni et al., 2008; Lee et al., 2023).

Additional proposed mechanisms include the attenuation of mitochondrial dysfunction, a direct positive inotropic effect, and inhibition of nitric oxide release (Lee and Sohn, 2023). Bupropion has been shown to increase reactive oxygen species and mitochondrial death (Ahmadian et al., 2017). LE may suppress the generation of the reactive oxygen species and mitigate mitochondrial dysfunction mediated cardiotoxicity. In a rat model, LE has also been shown to inhibit nitric oxide release and increase left ventricular systolic pressures (Shin et al., 2014). An increase in vascular resistance via reduction in endothelial nitric oxide production may help to reverse bupropion-induced cardiovascular collapse (Lee and Sohn, 2023).

These theories can explain why LE therapy is effective in drugs that are highly lipid soluble (Sirianni et al., 2008; Lee et al., 2023). Therefore, there was scientific plausibility that utilizing LE therapy to draw the medication into the vascular space would augment the effectiveness of the plasma exchange in removing it from the circulation. Bupropion is highly lipophilic, having a lipid/aqueous partition coefficient (log P 3.47) that is comparable to that of bupivacaine (log P 3.64) (Sirianni et al., 2008). In looking at the effects of LE therapy with amitriptyline, another similarly lipophilic drug, when the lipid emulsion was given 30 min after a toxic amitriptyline dose, the amitriptyline concentration in the heart and brain decreased while that in the arterial plasma increased, suggesting that the lipid emulsion mediated partitioning from organs with high blood flow to the arterial blood (Heinonen et al., 2013). This provides a plausible mechanism, where the LE therapy can be used to shift the drug into the plasma compartment and then use the plasma exchange to remove it, given that the protein binding is significant.

With therapeutic dosing, the reported elimination half-life of bupropion and hydroxybupropion is approximately 21 and 20 h, respectively. In the setting of massive overdoses, the pharmacokinetics are likely altered secondary to saturated metabolic pathways, poor intestinal perfusion, pharmacobezoar formation, and renal and hepatic insufficiency (Jefferson et al., 2005; Schmit et al., 2017). In our patient, urine bupropion and hydroxybupropion levels prior to the first plasma exchange were 119,000 ng/mL and 89,200 ng/mL, respectively. After the first plasma exchange, urine levels dropped to 20,900 ng/mL and 19,300 ng/mL, respectively, an 82% decrease in bupropion and 78% reduction in hydroxybupropion levels. Although there are no reports on the correlation between serum and urine bupropion and hydroxybupropion levels, assuming creatinine adjusted urine concentrations decrease in a proportional fashion to serum, this reduction would exceed expected elimination without extracorporeal clearance modalities. As shown in Table 1, the reduction in urine levels continued throughout the duration of the plasma exchanges. Of note, due to variability in the timing of some urine samples not allowing for redistribution and potential urinary equilibration to serum levels, there was an increase in urine levels for two of the plasma exchanges.

In conjunction with the reduction in urine drug levels, there was noted significant improvement in the patient’s neurologic and cardiovascular condition during this treatment period. Echocardiogram revealed improvement in ejection fraction from 18% to 33% following the first plasma exchange, while simultaneously the patient’s encephalopathy improved. Within hours following the first LE-TPE therapy, the patient required initiation of sedation due to purposeful movements after demonstrating no improvement in her encephalopathy prior to the initiation of LE-TPE therapy.

While some of the post-exchange urine drug levels increased from pre-exchange throughout the treatment course, this likely can be explained by a few factors. The pre-exchange LE therapy may transiently increase plasma concentrations via drug migration to the central compartment and potential transient increase in absorption of remaining drug in the gastrointestinal tract. Early urine sample timing post-exchange may reflect the transient period of elevated serum levels and potential lagging urine concentration equilibration (Perichon et al., 2013; Shi et al., 2013). Urine samples with adequate time post-exchange showed a consistent downtrend apart from exchange two, in which the post-exchange hydroxybupropion level increased while bupropion fell. This is likely a reflection of the patient’s improving metabolic function as hydroxybupropion levels in therapeutic doses are 5–10-fold higher than bupropion levels (Costa et al., 2019). Initial impaired hepatic function or saturated cytochrome metabolism would compromise the ability to metabolize the parent compound, but with clinical improvement, hydroxybupropion levels began to predominate. If solely examining the pre-exchange levels, there remains a clean and consistent downtrend of bupropion and hydroxybupropion levels with the exception of the eventual hydroxybupropion predominance described above.

The concomitant metoprolol ingestion, while contributing to the overall presentation, likely benefited little from the intralipid/plasma exchange therapy, given unclear benefit of LE therapy and pharmacokinetics not conducive to removal via plasma exchange (Petersen et al., 2019; Zamir et al., 2022). Therefore, the significant clinical improvement seen with our patient hours after initiation of the combined LE and TPE were likely due to the effect of these therapies on bupropion and hydroxybupropion levels, as the clinical improvement happened simultaneous to the drop in the bupropion and hydroxybupropion drug levels. One potential limitation of this report includes the fact that the plasma exchanges were performed by a highly experienced apheresis team, especially in performing plasma exchange procedures in critically ill patients. The experience of this team mitigates some of the risks of the plasma exchange. This may limit the generalizability of this intervention if this resource is not available.

## 4 Conclusion

This report describes the effect of LE therapy followed by plasma exchange for five consecutive days following a massive bupropion XL overdose resulting in cardiac arrest following severe cardiogenic shock. In addition to the rapid clinical improvement of the patient with the initiation of this therapy, this report describes the effect of the paired lipid emulsion therapy and plasma exchange on the urinary elimination of bupropion and hydroxybupropion in the setting of a massive overdose.

## Data Availability

The raw data supporting the conclusions of this article will be made available by the authors, without undue reservation.
